# Dissecting the Molecular Features of Systemic Light Chain (AL) Amyloidosis: Contributions from Proteomics

**DOI:** 10.3390/medicina57090916

**Published:** 2021-08-31

**Authors:** Paola Rognoni, Giulia Mazzini, Serena Caminito, Giovanni Palladini, Francesca Lavatelli

**Affiliations:** 1 Amyloidosis Research and Treatment Center, Fondazione IRCCS Policlinico San Matteo, V.le Golgi 19, 27100 Pavia, Italy; g.mazzini@smatteo.pv.it (G.M.); serena.caminito01@universitadipavia.it (S.C.); giovanni.palladini@unipv.it (G.P.); 2 Department of Molecular Medicine, University of Pavia, Via Forlanini 6, 27100 Pavia, Italy

**Keywords:** amyloidosis, immunoglobulin light chains, protein misfolding, amyloid fibrils, post-translational modifications

## Abstract

Amyloidoses are characterized by aggregation of proteins into highly ordered amyloid fibrils, which deposit in the extracellular space of tissues, leading to organ dysfunction. In AL (amyloid light chain) amyloidosis, the most common form in Western countries, the amyloidogenic precursor is a misfolding-prone immunoglobulin light chain (LC), which, in the systemic form, is produced in excess by a plasma cell clone and transported to target organs though blood. Due to the primary role that proteins play in the pathogenesis of amyloidoses, mass spectrometry (MS)-based proteomic studies have gained an established position in the clinical management and research of these diseases. In AL amyloidosis, in particular, proteomics has provided important contributions for characterizing the precursor light chain, the composition of the amyloid deposits and the mechanisms of proteotoxicity in target organ cells and experimental models of disease. This review will provide an overview of the major achievements of proteomic studies in AL amyloidosis, with a presentation of the most recent acquisitions and a critical discussion of open issues and ongoing trends.

## 1. Introduction

The term “proteomics” encompasses a spectrum of technologies, ultimately centered on protein analysis by mass spectrometry (MS), which allow characterizing the protein constituents of a sample of variable complexity, so called “proteome”. In the field of human pathology, amyloidosis is a prominent example in which proteomics has had a significant impact in the clinical routine and in the research context, providing important contributions for a better knowledge of the disease mechanisms and for improving the diagnostic process [1,2]. The wide use of proteomics in amyloidoses relates to the fact that altered proteins are the key pathogenic players in these pathologies. In fact, the characteristic feature of amyloidoses is the extracellular deposition of insoluble amyloid fibrils, elongated polymers with a cross-β structure derived from the misfolding and polymerization of autologous proteins. Amyloid formation and deposition ultimately leads to tissue damage and dysfunction of the affected organs [3,4]. Amyloidoses are classified according to the precursor protein and constitute a group of diseases heterogeneous for pathogenesis and clinical manifestations. To date, approximately 40 different proteins are identified as amyloidogenic in humans [5].

Currently, the most common form of systemic amyloidosis in Western countries is AL (amyloid light chain) amyloidosis, which has an incidence of about 10 cases per million persons/year [6,7]. This disease is caused by deposition of free monoclonal immunoglobulin light chains (LCs), produced by a bone marrow plasma cell clone and transported through bloodstream to target tissues [3]. In AL amyloidosis, a multitude of proteomic approaches have been used in clinical and research contexts, providing a wealth of novel information on topics that span from definition of primary sequence of LCs and germline gene usage, to post-translational modifications (PTMs), up to changes in the physiology of affected cells and tissues. The proteomic methodologies and technology used in this field encompass a wide spectrum in terms of instrumentation and applications, ranging from more traditional gel-based methods, to LC-MS/MS-based analyses, up to the most recent MALDI imaging mass spectrometry studies. Importantly, various types of ex vivo relevant specimens have been the object of analysis by proteomics in AL amyloidosis, including serum [8,9,10,11], urines [12,13], formalin-fixed paraffin-embedded (FFPE) [14,15,16,17,18,19] tissues, or fresh/frozen specimens such as subcutaneous abdominal fat [15,20,21,22]. This review will provide a critical overview of how proteomics has expanded our molecular knowledge and opened new clinical perspectives in AL amyloidosis, with a brief description of the techniques and methodologies used for each analytical task, presentation of the most recent acquisitions and a critical discussion of open issues and ongoing trends.

## 2. AL Amyloidosis: An Overview

Light chain amyloidosis is a polymorphic disease, whose clinical manifestations depend on the pattern of deposition (systemic vs. localized) and on which organs are affected. In the systemic cases, multi-organ involvement is present in most patients at presentation. With the exception of the central nervous system, virtually every organ can be targeted by AL amyloid deposition, including heart, kidney, soft tissues, liver, gastrointestinal tract, autonomic and peripheral nervous system. In particular, heart involvement is frequent (75% of cases) and is a major prognostic factor [4], leading to death for chronic cardiac failure or fatal arrhythmias.

In systemic AL amyloidosis, monoclonal free LCs with a peculiar misfolding propensity are produced in excess by a usually small bone marrow plasma cell clone, and they misfold and aggregate into amyloid fibrils in the interstitium of target organs [7]. Immunoglobulin LCs are 22–23 kDa proteins consisting of two β-sheets-rich domains: the N-terminal variable domain (V_L_) and the constant one (C_L_) [23]. The V_L_ region is characterized by high sequence variability, due to gene recombination and somatic hypermutation during the protein maturation process, whereas C_L_ displays limited sequence variation within each of the two light chains isotypes, κ and λ [23,24,25]. Both λ and κ free LCs assemble into disulfide-linked homodimers [26,27]. The majority (approximately 75%) of all monoclonal amyloidogenic LCs belong to the λ isotype [4]. Noteworthy, only a small fraction of all possible monoclonal LCs form amyloid fibrils in vivo, and the majority of patients with monoclonal gammopathies do not develop amyloid deposits, despite high free LC concentrations for extended time. Features intrinsic to the LC’s primary sequence are likely related to pathogenicity; however, given the high sequence variability (which translates in the fact that each LC is virtually unique), dissecting the molecular bases of LC aggregation and identifying the factors linked to misfolding and to organ tropism are challenging tasks. Recent high-resolution structural analyses have elucidated important details regarding the assembly of AL amyloid aggregates in vivo. Visualization of cardiac amyloid fibrils using cryo-electron microscopy, in particular, has shown that LCs derived from different germlines aggregate in fibrils with distinct structure; however, these share the feature that the rigid core of the fibril is always composed by the variable domain of the LC, in which the disulfide bridge is conserved and which undergoes complete unfolding of the native structure prior to adopting the alternative fibrillar conformation [28,29,30].

The process of LCs amyloid formation is associated with tissue damage and organ dysfunction, through a complex interplay of mechanisms. Whereas the presence of fibrillar aggregates alters the composition of the extracellular space and the tissue mechanics, also pre-fibrillar LCs are considered to contribute to damage in this form of amyloidosis, through direct proteotoxicity [31,32]. This concept derives from the clinical evidence that variation in the degree of organ dysfunction (especially in relation to the heart, whose sufferance can be monitored using the circulating biomarkers brain natriuretic peptide (BNP), N-terminal proBNP and Troponins) occurs simultaneously with variations in the serum concentration of amyloidogenic LCs, even in the absence of detectable changes in amyloid deposits [33,34,35,36]. In light of these observations, experimental studies have been performed to explore the direct proteotoxicity of amyloidogenic LCs, confirming that these species are harmful to cultured target cells and small animal models, even in the absence of fibrils [37,38,39,40,41,42,43,44,45,46].

Given the fact that deposition of amyloid fibrils, and hence organ deterioration, are progressive, early disease detection and accurate definition of the amyloidosis type, i.e., identification of the protein responsible of amyloid formation, are critical for establishing diagnosis and defining the appropriate therapy. Indeed, a thorough understanding of the bases of misfolding and proteotoxicity of LCs, as well as identification of the factors conferring greater risk to develop amyloid deposits from monoclonal gammopathies of undetermined significance (MGUS) or to drive LC deposition in vital organs such as the heart [47,48], are essential in the perspective of progressing into accurate early diagnosis, advancing the therapeutic possibilities and ameliorating prognosis. The following paragraphs will illustrate the contribution of proteomics in different areas, from the characterization of the amyloidogenic LC itself, to the elucidation of the perturbations occurring in target tissues and cells.

## 3. Amyloidogenic Light Chains: Biological Impact and Clinical Implications of Proteomic Characterization

In recent years, MS has been increasingly used to investigate the features of amyloidogenic and fibrillary LCs. Due to the uniqueness of each LC’s sequence, predicting its primary structure from the genome is impossible [49]. Traditionally, LC sequencing has been achieved by molecular biology tools, upon isolation and cloning of its coding mRNA from the bone marrow plasma cell clone [50,51,52,53,54,55]. However, advancements in MS instrumentation, proteomic techniques and reagents, availability of annotated databases and novel bioinformatics tools have had a profound impact in this regard. Nowadays, an unprecedented wealth of data on immunoglobulin LCs sequences can be obtained from the direct proteomic analysis of these proteins from amyloid deposits or biological fluids. In terms of throughput, speed and sensitivity, proteomics also favorably compares with traditional biochemical methods such as Edman degradation [10,18,20,21,56,57,58] for sequencing LCs extracted from amyloid fibrils.

Over the years, several reports have been published on the proteomic analysis of monoclonal LCs in AL amyloidosis. The field has been opened by pivotal studies that utilized a combination of chromatographic and MS techniques to obtain detailed sequence characterization and locate PTMs [13,15,59] in deposited LCs from single patients. More recently, also thanks to the use of laser-microdissection (LMD)-based methods to enrich amyloid deposits from biopsy specimens, MS analysis of LC fibrils has been performed on a patients-population scale [18,60,61,62]. In LMD-MS, the Congo red-positive areas in a tissue section are dissected using a laser beam, collected and analyzed by mass spectrometry, allowing to enrich the signal from amyloid proteins over the tissue background [14]. In a recent study, Mayo Clinic researchers reported the proteomic analysis and typing of amyloid in a large cohort of more than 16,000 cases, more than half of which were AL type [18].

Using annotated databases and dedicated bioinformatics, tissue proteomics data have been used to assign the gene family and germline genes used by deposited LCs [47]. The throughput of this approach and the high number of analyzed samples allowed obtaining extensive protein-based information without the requirement for bone marrow material, and well representative of the population of patients with systemic and localized AL amyloidosis. Although proteomics, in contrast with gene sequencing, usually provides sequence information only for limited regions of the protein (the peptide ions visible during the MS analysis), alignment with annotated LC sequence databases provides a significant chance to assign the pertinent germline/gene family. These MS-based data have provided novel information on germline usage in AL and its relation with specific clinical features, such as organ tropism, severity of damage, disease prognosis and other characteristics of the underlying monoclonal gammopathy. These studies, in particular, confirmed that more than half of all amyloidogenic LCs derive from the rearrangement of a small subset of germline donors (λ1, λ2, λ3, λ6 and κ1) [47,51], and validated the association of specific genes with tropism for certain organs. In fact, *IGLV6-57* patients are more likely to have renal involvement, whereas *IGLV1-44* germline associates with cardiac, *IGLV2-14* with peripheral nerves and *IGKV1-33* with liver involvement [47,51,55]. Immunoglobulin LC germline gene usage was also found to differ between IgM and non-IgM AL amyloidosis, both in the κ and λ families, possibly contributing to explain the distinct clinical presentation of IgM vs. non-IgM forms [60]. Proteomics-determined immunoglobulin germline gene usage was also suggested to have prognostic significance in patients who did not achieve VGPR or better to initial chemotherapy [48]. From a practical point of view, routine definition of the LC subtype during proteomic analysis of patients’ specimens can provide information useful to tailor the clinical management, for example to closely monitor the development of cardiac deposits in patients with LCs belonging to specific classes.

Also circulating and urinary amyloidogenic LCs have been studied using proteomics. Free LCs are frequently present in the urines of patients with AL amyloidosis and multiple myeloma, and can be used as a source of material for LC characterization and for in vitro experimental studies [13,63,64,65]. Proteomic study of serum LCs, in contrast, is hampered by the complexity of this matrix and by the background of polyclonal and non-involved immunoglobulins. Most high-throughput methods for MS analysis of serum amyloidogenic LC have been described only recently, and usually require careful upfront purification strategies. In most cases, these consist in immunoenrichment steps using bead-bound specific antibodies or nanobodies. A first approach specifically dedicated to proteomic analysis of serum free LCs was developed by our group and is based on immunopurification of these species using an optimized bead-based approach [8]. Under non-reducing conditions, the purified material does not contain intact immunoglobulins, contains only small amounts of polyclonal free LCs, and is suitable to study amyloidogenic proteins by gel-based and gel-free proteomics. 

Recently, new LC-MS (Liquid Chromatography-Mass Spectrometry) and MALDI-TOF (Matrix Assisted Laser Desorption Ionization-Time Of Flight) MS-based approaches specifically dedicated to clinical analysis of serum LCs and free LCs have been described [66,67,68,69,70,71,72]. A major such example is the method implemented by Mayo Clinic investigators, known as MASS-FIX (based on immunoenrichment of immunoglobulins and LC subclasses, followed by MALDI-TOF MS), for the detection and evaluation of monoclonal components in plasma cell dyscrasias, including AL amyloidosis [9,10,73,74,75,76]. The favorable features of this approach in terms of diagnostic sensitivity and specificity supported its rapid translation into the clinical routine. 

An important aspect in studying amyloid LC precursors by MS concerns the chance to characterize the PTMs that occur on these proteins in vivo, a crucial aspect to elucidate the possible factors behind stability loss and fibril deposition. Diverse chemical modifications affecting amyloidogenic LCs have been described over the years through multiple biochemical approaches, such as oxidation [8,63,77], cysteinylation [12,59], deamidation [78] and glycosylation [9,10,73,74,75,79]. Glycosylation is especially interesting in relation to AL amyloidosis, because recent reports, based on MASS-FIX-based population-based analysis of serum samples from individuals with various monoclonal gammopathies, support the concept that this PTM may be related to a LC’s ability to form amyloid deposits [49]. In fact, MASS-FIX showed that peaks in the MALDI-TOF mass spectrum, corresponding to glycosylated LCs, are present in AL patients with a statistically higher frequency compared to MGUS or MM [9,10,73,74,75]. In fact, up to 33% of AL-κ and 10.2% of AL-λ patients show a pattern consistent with glycosylation, compared with 3.7% and 4.9% of patients with κ and λ non-AL plasma cell disorders [9,79]. Importantly, in patients who develop AL amyloidosis, N-glycosylation of LCs was demonstrated to be present from the time of diagnosis of monoclonal gammopathy of undetermined significance (MGUS), and represents an independent risk factor for MGUS progression to AL amyloidosis and other plasma cells disorders [73,75]. Besides opening important perspectives on the biochemical bases of LC amyloidogenesis, these data show that monitoring LC glycosylation by MS has relevant diagnostic and prognostic clinical implications. In fact, MS analysis allows identifying a subset of MGUS patients at higher risk of progression to AL, in whom the follow-up and diagnostic workup can be tailored, with the goal to achieve early diagnosis and potentially reduce morbidity and mortality. 

## 4. The AL Amyloid Proteome: Disease Typing, Characterization of Deposited LC Proteoforms and the Amyloid Environment

The advent of proteomics has been a revolution in the field of amyloidosis typing, because it allows identifying the deposited proteins independently from the use of antibodies, through alignment of experimental tandem mass spectra against protein sequence databases. This type of analysis is routinely performed in major amyloid centers, and proteomic data from thousands of AL cases are now available [16,18,20,61,62,80,81]. Proteomic typing offers important advantages that have been extensively reviewed elsewhere [61,82]; however, AL amyloidosis also poses unique challenges to this approach. In fact, the above-discussed uniqueness of sequence in the V_L_ translates in the fact that protein databases cannot contain the full sequence of each monoclonal LC, and therefore peptide ions from the variable domain, which forms the structured core of the fibrils, may not be matched [21,28,83]. In some instances, this may qualitatively and quantitatively affect protein identification and impair typing. It is however a merit of proteomics to have provided also strong confirmatory evidence that AL deposits invariably contain peptides from the LC’s constant domain, indicating that the full length protein takes place in fibril deposition [21,84]. Identification of these peptides is not affected by sequence variability, and often aids in typing. 

In light of the above considerations, the availability of annotated databases with a large number of verified LC sequences acquires growing importance. Besides in-house augmented databases used in some centers, other resources such as AL-Base [85] are available to the whole scientific community, and represent important instruments to improve the performances and reproducibility of diagnostic proteomics of AL amyloidosis. In addition to these sequence-related issues, the presence of PTMs affecting the long-lived deposited LCs should not be ignored. The wide number of chemical modifications (e.g., deamidation, truncation) [21] known to affect amyloid LCs may in fact impact protein identification and peptide matching. These natural modifications sum up to the chemical ones that may occur as a result of sample fixation and paraffin embedding, such as the formation of methyl lysines [58]. 

An especially important chapter regarding PTMs of fibrillary LCs concerns proteolysis, which translates in the presence of a complex population of fragments in the deposits [21,84,86]. Truncation translates into formation of semi-tryptic peptides, and database search should be tailored to account for their presence. The pathogenetic role of proteolysis in AL (i.e., whether it precedes or follows LC deposition) is still debated, and the discussion of this topic is beyond the scope of this review. Indeed, new findings on the features of amyloid LC fragments come from a recent proteomic study performed by our group, dedicated to the investigation of the *termini* of these proteoforms [87] in ex vivo cardiac deposits. In particular, the N- and C-terminal residues of fragments were chemically derivatized, and the labeled residues were identified by LC-MS/MS and bioinformatics [87]. The data showed that fragmentations mainly occurs in the C_L_; the cleavage sites that generate LC fragments are mostly located in poorly structured regions of the fibrillar structure, suggesting that the ensemble of proteolytic events observed in mature ex vivo fibrils largely reflects extensive proteolytic remodeling of the preformed aggregates [87].

Amyloidogenic LCs, however, are not the only molecular species present in the deposits. In all amyloidoses, the aggregates are indeed complex assemblies with an intricate biochemical composition. The proteomic analysis of ex vivo tissues has provided relevant information in this respect [14,18,20,57,80,88]. Besides the main fibrillar precursor, other blood-borne proteins and specific tissue proteins (so-called “amyloid protein signature”, or “amyloid-associated proteins”) are commonly associated with fibrils, regardless of the amyloidosis type [21,22,57,88,89]. In addition, other molecules, such as glycosaminoglycans and lipids, co-localize with aggregates [3,90]. Although the physico-chemical relation between fibrils and amyloid-associated components is not yet defined, in vivo amyloid deposits are better described as an admixture of molecules, rather than simply as fibrils of a defined protein. A recently proposed classification system categorizes the amyloid-proteome proteins into four functional categories: fibrillary proteins found in the patient; potential fibril-forming proteins found in other types of amyloid; non-fibril proteins, including some being amyloid signature proteins [91]. Assessment of whether amyloid composition relates to organ involving, to amyloidosis type and to disease severity would be of major interest for further understanding the molecular bases of these diseases [18,21].

Proteins typically found in amyloid deposits include, among others, serum amyloid P (SAP), apolipoprotein E (ApoE), apolipoprotein AIV (ApoAIV) [22], vitronectin (VTN) [92], complement and clusterin [93]. The fact that these proteins are “universal” amyloid components has led to suggest that the concomitant presence of several of these species in the proteome map of a tissue could be used as an “amyloid proteome signature”, useful for diagnostic purposes [22,88,89]. In fact, identification of at least two among ApoE, SAP and ApoAIV was proposed as a mean to discriminate between amyloid-positive and amyloid-negative samples, and is often used as a surrogate to indicate that the sample contains amyloid fibrils. 

Importantly, however, recent proteomic analyses are also showing that quantitative and qualitative differences exist in the composition of the amyloid milieu across amyloidosis types—and possibly across deposition sites. Such differences were first documented by analyzing unfractionated subcutaneous periumbilical fat [57] using Multidimensional Protein Identification Technology (MudPIT) (an analytical approach that combines multi-dimensional chromatography with LC-MS/MS, in order to achieve optimal separation and thorough identification of a large number of distinct proteins in a complex sample). In this study, complement C3 was overrepresented in transthyretin-related amyloidosis (ATTR) compared to AL, whereas basal membrane-specific heparan sulfate proteoglycan-2 was increased in the latter. In a more recent report, LMD-MS was employed to compare proteins present in microdissected cardiac amyloid deposits between ATTR and AL amyloidosis [19]. The analyses showed that fibrils from cardiac AL patients have a less complex proteome composition, and confirmed that this form is characterized by a lower abundance of complement proteins compared to ATTR, whereas the extracellular chaperone clusterin was found to be overrepresented in AL kappa patients compared to the other types [19].

Additional important knowledge is also being provided by another proteomic approach, MALDI imaging mass spectrometry (IMS). This technology possesses the unique capability of allowing direct MS analysis of histology sections, thereby preserving the spatial distribution of proteins [92,94,95]. MALDI-IMS studies allow demonstrating the co-localization of amyloid-associated proteins with Congo Red-positive areas, but are also disclosing differences in the peptides with which amyloid-associated species are identified in different amyloidosis types and tissues. The MALDI-IMS distribution of peptides from a set of amyloid-associated proteins (e.g., ApoE, SAP, VTN) has in fact been proposed as part of a possible strategy for amyloid typing [94], since specific peptide ions create a type and organ specific MALDI-IMS signature [95]. These observations may relate to distinct conformation, abundance or localization of these molecular species, opening unprecedented questions and new perspectives in the study of amyloidoses. 

Finally, a specific chapter in which proteomics has provided new relevant insights is represented by localized AL amyloidosis. In these cases, which represent the majority of all localized amyloidoses, fibril deposition is focal and limited to the anatomical site where LC overproduction occurs. Localized AL has long represented a puzzling entity from the pathogenetic point of view, both in terms of nature of LCs-producing cells and of the pathogenic LC itself. Although some organs, such as lymph nodes or lung, can be affected by localized deposits or be involved in the context of systemic disease, truly localized AL may indeed represent a unique form of LC deposition, clinically and biochemically. Recent proteomic analyses support this concept, showing that localized AL is peculiar in terms of germline gene usage and heavy chains co-deposition (which is more common in localized than in systemic cases) [47,48]. The distribution of immunoglobulin LC germline genes and families for patients with localized AL is similar to that seen in the normal B-cell repertoire, with IGLV6 found less commonly than in systemic AL and the IGKV3 family more commonly (*IGKV3-20* gene in particular). In addition, IGKV and IGLV gene family usage also appears to have an organ-specific distribution, with IGKV3 and IGLV2 being most prevalent in lung, and IGKV1 and IGLV3 in skin. Notably, the discovery that multiple LC variable family members co-exist in the deposits of some localized amyloidosis cases (eg, laryngotracheal amyloidosis) suggests that the process of amyloid deposition in these instances is nonclonal [96]. 

## 5. Proteomic Contributions to Deciphering the Molecular Bases of Cell and Organ Damage in AL Amyloidosis

Clinical evaluation of organ dysfunction biomarkers [4,7] and experimental evidence indicate that tissue damage in AL amyloidosis is not only due to fibril deposition, but also to pre-fibrillar amyloidogenic LCs, which are themselves directly toxic for target cells [3,31,32,36,38]. Given the fact that cardiac involvement is a major prognostic factor in AL amyloidosis [31], a great deal of experimental work has been devoted to establishing systems suitable to reproduce in vitro LC-mediated damage in the heart. Through the analysis of amyloid-affected human tissues and disease models, proteomics has indeed provided important contributions for understanding the molecular perturbations associated with AL amyloid deposits or with exposure to amyloidogenic LCs. 

Using both gel-based and shotgun proteomic techniques, our group explored the global proteome changes in adipose tissue biopsies from AL amyloidosis patients [57]. The analyses were performed without tissue fractionation, thus allowing to study not only the amyloid areas, but also cellular proteins and the extracellular space. These investigations demonstrated that the presence of AL fibrils is associated with changes in the abundance of a specific subset of tissue proteins, which are expressed by cells and are normal constituents of subcutaneous fat. The main altered compartments include the protein folding apparatus (with reduction in levels of intracellular proteins such as heat shock 70 kDa protein 6, alpha-crystallin B chain and serpin H1), extracellular matrix and basal lamina, and the machinery involved in various metabolic pathways of adipocytes (including mitochondrial respiration, lipid metabolism and glycolysis) [21,80]. These studies indicate, on one side, that extracellular protein misfolding and deposition translates into intracellular change in tissue resident cells. On the other side, remodeling of the extracellular space, with increase in collagen and heparan sulfate proteoglycans and decrease of the less amyloidogenic keratan sulfate and anti-amyloidogenic laminins, may determine a pro-amyloidogenic environment, potentially directing the soluble amyloid precursors into the pathway of fibril formation. Tens of other proteins involved in a variety of actions were altered in AL-affected fat tissue, whose targeted study may cast new light on the pathogenesis of this disease, or which could be investigated as possible biomarkers. 

A peculiar composition of the local tissue proteome in AL patients has also been documented through the analysis of microdissected amyloid areas in the heart [19]. Compared to unaffected controls, matrix related proteins (COL1A1, COL1A2, COL3A1, and TIMP3), matrix-remodeling proteins (MUC19, PRELP, and PRG4), proteins involved in enzymatic processes (GPD1 and PIK3C3), and SERPINE2 were found to be elevated in patients. Differences were also documented between κ and λ cases, with the formers having higher levels of clusterin (CLU), prolargin (PRELP), and SERPINE2. AL patients shared some proteomic features with ATTR ones, but also presented some notable differences. In particular, both forms showed increased abundance of proteins involved in matrix remodeling and enzymatic processes; however, the proteome of ATTR samples was enriched in contractility proteins, while cardiac AL deposits showed increased levels of keratins [19], suggesting different mechanisms of organ damage between amyloidosis types.

Regarding in vitro studies, both cell culture systems (human and rodent cardiac cells [37,39,40,41,42,43,44,45]), and simple animal models (*C. elegans* and zebrafish [38,41,46]) have been used to define the molecular bases of damage caused by soluble, pre-fibrillar precursors [37,38,39,40,41,42,43,44]. These experimental models share an important feature: the observed damage is exerted only by LCs that are cardiotropic in patients, and not by amyloidogenic LCs that target other organs or by non-amyloidogenic LCs. Exposure to cardiotropic LCs leads to functional and cellular alterations, including impairment in viability, oxidative stress, mitochondrial damage, lysosomal dysfunction and impaired autophagy [37,38,39,40,42]. Proteomic studies, combined with cell-based and functional assays, significantly contributed to exploring the bases of LC-mediated damage in these models [45,97,98]. 

Using an interactomic approach based on co-immunoprecipitation coupled with MS and bioinformatic analyses, our group showed that amyloidogenic cardiotoxic LCs interact with a subset of cellular proteins in vitro, mainly localized in the mitochondria and involved in viability and metabolic functions [45]. Following this evidence, imaging studies on primary human cardiac cells cultures (fibroblasts, hCF) confirmed that cardiotoxic LCs are internalized, co-localize with mitochondria and interact with specific mitochondrial proteins (VDAC, OPA1 and ACAD9). Concomitantly, ultrastructural changes (cristae widening) in these organelles were documented, suggesting that exposure to cardiotropic LCs ultimately affects the functionality of these organelles [45]. Notably, cardiotoxic LCs interact with mitochondria exclusively in cardiac fibroblasts and not in dermal ones, indicating that LC toxicity, in parallel with amyloid deposition, has a tropism for the specific target cells [45].

The perturbations in hCF after exposure to amyloidogenic LCs were also explored by studying their cell proteome. Thanks to a combination of two-dimensional differential in-gel electrophoresis (2D-DIGE) and label-free high-resolution MS shotgun analysis [97], several proteins were found to be significantly altered upon exposure to cardiotropic LCs, including proteins involved in viability, cytoskeletal organization, protein quality control apparatus, apoptosis and mitochondrial function [97]. 

## 6. Perspectives

AL amyloidosis poses specific challenges to clinicians and researchers. From a molecular point of view, the uniqueness of sequence of the precursor LC translates in the fact that each monoclonal protein is biochemically and biophysically distinct, making it complex to find common misfolding determinants and unifying features that explain proteotoxicity. The sequence peculiarity is a challenge also for proteomics and requires a significant collective effort to create curated LC databases containing large numbers of patients-derived sequences, and dedicated bioinformatics tools. The presence of PTMs is an additional factor that augments the biochemical complexity of LC fibrils, long-lived structures that undergo important remodeling during their permanence in tissues.

The last decade has indeed witnessed an epochal change in the diagnostic possibilities, clinical management and molecular understanding of AL amyloidosis, and proteomics has been an important protagonist of this change. MS has gained a primary role in the clinical management of AL, not only for typing the amyloid deposits, but also to characterize the circulating precursors. In the diagnostic process, the transition from traditional methods to proteomics-based ones is a reality that is expected to last. The information provided by proteomics on the tissue alterations associated with LC deposition is invaluable to understand organ damage at the molecular level, possibly driving the design of novel therapeutic approaches and the discovery of potential biomarkers.

Given the versatility of the approach, dedicated proteomic studies may provide future information also on additional and still underexplored aspects of the disease, such as the nature of LC oligomeric aggregates, or the molecular relations between the components of the amyloid deposits. Will structural information be the next frontier to conquer for proteomics in AL amyloidosis and other amyloid diseases?

## Data Availability

Not applicable.
